# The Robust Weighted Multi-Objective Game

**DOI:** 10.1371/journal.pone.0138970

**Published:** 2015-09-25

**Authors:** Shaojian Qu, Ying Ji, Mark Goh

**Affiliations:** 1 Business School, University of Shanghai for Science and Technology, Shanghai, P.R. China; 2 The Logistics Institute-Asia Pacific, National University of Singapore, Singapore, Singapore; 3 Natural Science Center, Harbin Institute of Technology, Harbin, P.R. China; 4 NUS Business School, National University of Singapore, Singapore, Singapore; Nankai University, CHINA

## Abstract

This paper studies a class of multi-objective *n*-person non-zero sum games through a robust weighted approach where each player has more than one competing objective. This robust weighted multi-objective game model assumes that each player attaches a set of weights to its objectives instead of accessing accurate weights. Each player wishes to minimize its maximum weighted sum objective where the maximization is pointing to the set of weights. To address this new model, a new equilibrium concept-robust weighted Nash equilibrium is obtained. The existence of this new concept is proven on suitable assumptions about the multi-objective payoffs.

## 1 Introduction

This paper addresses the following multi-objective game with finite players in the normal form, *MG* : = (*N*, {*S*_*i*_}_*i* ∈ *N*_, {*F* ^*i*^}_*i* ∈ *N*_). Here *N* : = {1, ⋯, *n*} is a finite set of players, $S_{i}\subset R^{a_{i}}$ is the set of actions for player *i* and *F* ^*i*^ is the multi-objective payoff function of player *i* and the number of objectives is set as *b*_*i*_. Then *F* ^*i*^ is defined as a mapping from the Cartesian product $S:=\prod_{i\in N}S_{i}$ into $R^{b_{i}}$ (since this paper considers the multi-objective game, so without any loss of generality throughout this paper we assume that each player *i* has more than one objective in his payoff function, that is *b*_*i*_ ≥ 2). Given *x* ^*i*^ ∈ *S*_*i*_ to be played, we specifically define $x^{i}:={\left(x_{1}^{i},\cdots ,x_{a_{i}}^{i}\right)}^{T}\in R^{a_{i}}$. In this paper, given *x* : = (*x* ^1^, ⋯, *x* ^*n*^) ∈ *S* to be played, then each player *i* has a multi-objective payoff function as $F^{i}(x):=(f_{1}^{i}(x),\cdots ,f_{b_{i}}^{i}(x))$ which means that each player *i* has more than one competing objective.

Since there is more than one objective for each player in the above multi-objective game, an accepted equilibrium concept is the Pareto equilibrium, which has already been used in [1] and can be defined as follows.

**Definition 1.1** *A mixed strategy* *x* ∈ *S* *is a Pareto (resp. weak Pareto) equilibrium for MG if for each player* *i*, *x* ^*i*^ ∈ *S*_*i*_ *is a Pareto optimal strategy (resp. a weak Pareto optimal strategy) against* *x*, *i.e., for any player* *i*, *i* ∈ *N*, *whose strategy* *x* ^*i*^ ∈ *S*_*i*_ *to the other player’s strategies* $x^{-i}\in S_{-i}:=\prod_{j\in N,j\neq i}S_{j}$ *is a Pareto optimal strategy (resp. a weak Pareto optimal strategy) respectively to the following multi-objective optimization,*

$$\begin{array}{c} \min_{x^{i}\in S_{i}}F^{i}\left(x\right), \end{array}$$ (1.1)

*that is there is no strategy* *u* ^*i*^ ∈ *S*_*i*_ *such that*

$$\begin{array}{c} F^{i}\left(x^{-i},x^{i}\right)⪯F^{i}\left(x^{-i},u^{i}\right)\left(resp.\;F^{i}\left(x^{-i},x^{i}\right)≺F^{i}\left(x^{-i},u^{i}\right)\right), \end{array}$$

*where the partial orders ≼ and ≺ are defined as, for any given* *r*, *v* ∈ *R* ^*m*^,

$$\begin{array}{ccc} & & v≻r\left(resp.\;v≺r\right)⟺v-r\in R_{++}^{m}\;\left(resp.\;r-v\in R_{++}^{m}\right)\\ & & v⪰r\left(resp.\;v⪯r\right)⟺v-r\in R_{+}^{m}and\;r\neq v\left(resp.\;r-v\in R_{+}^{m}and\;r\neq v\right). \end{array}$$

MG is a generalization of the scalar criterion games and is used to modelling situations where two or more decision makers, called players, take actions by considering their individual multiple objectives. The study for multi-objective games dates back to Blackwell’s work which considers zero-sum games with multi-objective payoffs [2]. Since then, much attention has been siren to game models with multiple payoffs [3, 4]. One reason is that multi-objective models are more applicable to real-world situations [1].

In this paper, we consider *n*-person robust weighted MGs where each player has two or more objectives and is ambiguous about the weights to the objectives. Specifically, we focus on finite multi-objective games where the weights of objectives are uncertain and are assumed to belong to a convex and compact set. In our model, we assume that all players are risk-averse and each player uses a robust optimization approach to manage the uncertainty of weights, assuming that the other players are robust optimizers as well. Note that the robust optimization approach does not concern game data nor the other players’ strategies, but only the weights to the objectives.

In MGs, since there are several competing objectives to be considered for each player and it is not possible to simultaneously optimize all objectives, a commonly accepted approach for coping with this setting is the weighted approach. This approach assigns a nonnegative weight by considering the importance of the corresponding objective function. A player can then make a decision by optimizing a weighted sum objective by assuming that the other players also make their decision by optimizing their own weighted sum objective [1, 3, 5]. A weighted Nash equilibrium point can be obtained if each player makes a decision by optimizing his weighted sum function. However the current weighted approach has several shortcomings. As shown in applications, the weights are not known a priori and the player has to choose them. Ambiguity often exists in the choice of the weights to the objectives, as it is not easy to decide relative weights for each objective. In addition, as shown in the literature on multi-objective optimization the relative weights provided by the same decision-maker may rely on some elicitation methods [6, 7]. Therefore it is necessary to provide a new approach to cope with these issues.

Hence, our motivation to utilize a robust weighted approach is that it provides an alternative way to deal with ambiguity of the weights. Further, if each player under an MG chooses the robust weighted approach, then we show that there is at least a robust weighted Nash equilibrium which further guarantees the existence of the Pareto Nash equilibrium. As such that the primary contributions of this paper are as follows. We propose a robust weighted approach for multi-objective *n*-person non-zero sum games, extending the notion of robust weighted multi-objective optimization models to multi-objective games; Our work can also be seen as an extension of the robust one-shot scalar games. We prove the existence of a robust weighted Nash equilibrium.

## 2 Preliminaries

Suppose each player *i* has a set $W^{i}\subset W_{r}^{i}$ of weights for his objectives, then we can provide a robust weighted Nash equilibrium concept for MG, where $W_{r}^{i}:=\{w^{i}\in R_{+}^{b_{i}}|\;∥w^{i}{∥}_{1}=1\}$. We note that when *W* ^*i*^, ∀*i* ∈ *N*, reduces to a singleton, the robust weighted Nash equilibrium becomes the weighted Nash equilibrium.

**Definition 2.1** *Given a closed set* $W^{i}\subset W_{r}^{i}$, *i* ∈ *N*, *then a mixed strategy* *x* ∈ *S* *is a robust weighted Nash equilibrium of MG if for each player* *i*, *x* ^*i*^ *is the optimum to the following robust optimization problem*

$$\begin{array}{c} \min_{u^{i}\in S_{i}}\max_{w^{i}\in W^{i}}{\left(w^{i}\right)}^{T}F^{i}\left(x^{-i},u^{i}\right). \end{array}$$ (2.1)

For the simplicity, we denote by *RoP*(*W* ^*i*^) for given *W* ^*i*^ problem Eq (2.1) and its optimal value (with the convention that *RoP*(*W* ^*i*^) = +∞ if the problem is infeasible). For any *i* ∈ *N*, given two weight sets $W_{1}^{i}\subset W_{2}^{i}$, it is obvious that $\left(RoP(W_{1}^{i}))\leq (RoP(W_{2}^{i})\right)$. Furthermore in this robust weighted Nash equilibrium concept, although we do not suppose the convexity of *W* ^*i*^, ∀*i* ∈ *N*, actually the robust weighted Nash equilibrium concept of MG with closed weight sets combination $W:=(W^{1},\cdots ,W^{n})\subset W_{r}:=(W_{r}^{1},\cdots ,W_{r}^{n})$, is equivalent to the robust weighted Nash equilibrium concept of MG with closed weight sets *conv*(*W*) : = (*conv*(*W* ^1^), ⋯, *conv*(*W* ^*n*^)) ⊂ *W*_*r*_, where *conv*(⋅) denotes the convex hull. This is equivalent to showing that for any player *i* and fixed *x* ^−*i*^ ∈ *S*_−*i*_ the equivalence of the following two sets

$$\begin{array}{c} \chi_{i}=\{\left(\gamma ,x^{i}\right)\in R\times S_{i}|\;u^{i}\in S_{i},\;{\left(w^{i}\right)}^{T}F^{i}\left(x^{-i},u^{i}\right)\leq \gamma ,\;\forall w^{i}\in W^{i}\} \end{array}$$

and

$$\begin{array}{c} {\bar{\chi }}_{i}=\{\left(\gamma ,x^{i}\right)\in R\times S_{i}|\;u^{i}\in S_{i},\;{\left(w^{i}\right)}^{T}F^{i}\left(x^{-i},u^{i}\right)\leq \gamma ,\;\forall w^{i}\in conv\left(W^{i}\right)\}. \end{array}$$

It is easy from *W* ^*i*^ ⊂ *conv*(*W* ^*i*^) to see that for any $(\gamma ,x^{i})\in {\bar{\chi }}_{i}$, (*w* ^*i*^)^*T*^ *F* ^*i*^(*x* ^−*i*^, *u* ^*i*^) ≤ *γ*, ∀*w* ^*i*^ ∈ *W* ^*i*^, that is (*γ*, *x* ^*i*^) ∈ *χ*_*i*_, which means that ${\bar{\chi }}_{i}\subset \chi_{i}$. Next we are going to prove that $\chi_{i}\subset {\bar{\chi }}_{i}$. To this end, for any (*γ*, *x* ^*i*^) ∈ *χ*_*i*_, we show $(\gamma ,x^{i})\in {\bar{\chi }}_{i}$. For any *w* ^*i*^ ∈ *conv*(*W* ^*i*^), there exist *w* ^*ik*^ ∈ *W* ^*i*^, *k* = 1, ⋯, *κ*, such that $w^{i}=\sum_{k=1}^{\kappa }\lambda_{k}w^{ik}$, here *λ*_*k*_ ≥ 0(*k* = 1, ⋯, *κ*), and $\sum_{k=1}^{\kappa }\lambda_{k}=1$ for some *κ* ≤ *b*_*i*_ + 1. From (*γ*, *x* ^*i*^) ∈ *χ*_*i*_, (*w* ^*ik*^)^*T*^ *F* ^*i*^(*x* ^−*i*^, *x* ^*i*^) ≤ *γ*, *k* = 1, ⋯, *κ*, which implies that

$$\begin{array}{c} {\left(w^{i}\right)}^{T}F^{i}\left(x^{-i},x^{i}\right)=\sum_{k=1}^{\kappa }\lambda_{k}{\left(w^{ik}\right)}^{T}F^{i}\left(x^{-i},x^{i}\right)\leq \gamma . \end{array}$$

This means that $\chi_{i}\subset {\bar{\chi }}_{i}$. Therefore $\chi_{i}={\bar{\chi }}_{i}$, ∀*i* ∈ *N*. Thus, for the rest of this paper, we assume that *W* ^*i*^ is a closed and convex subset of $W_{r}^{i}$, ∀*i* ∈ *N*.

Different from the weighted Nash equilibrium, the robust weighted Nash equilibrium proposed in this paper seeks to find an equilibrium solution that is robust and that is feasible for the robust weight within the family of weights. Wang [1] shows that for any weighted Nash equilibrium of MG with weights given in $W_{r}^{i}$, ∀*i* ∈ *N*, then it is either a weak Pareto equilibrium or a Pareto equilibrium. In the following proposition, we show that the robust weighted Nash equilibrium of MG inherits the properties of the weighted Nash equilibria, that is any robust weighted Nash equilibrium of MG with weights set $W^{i}\subset W_{r}^{i}$, *i* ∈ *N* is either a weak Pareto equilibrium or a Pareto equilibrium.

**Theorem 2.2** *For any* *i* ∈ *N*, *given a closed set* $W^{i}\subset W_{r}^{i}$, if x* : = (*x* ^1*^, ⋯, *x* ^n*^) ∈ *S* *is a robust weighted Nash equilibrium of MG with the combination* *W* = (*W* ^1^, ⋯, *W* ^*n*^) *of weight sets, then we have the following results*,

1. x* *is a weak Pareto Nash equilibrium of MG;*
2. *if for any player* *i*, *i* ∈ *N*, *with all weights in* *W* ^*i*^ *are all positive, then* x* *is a Pareto Nash equilibrium of MG;*
3. *for any player* *i*, *i* ∈ *N*, if *x* ^i*^ *is the unique optimal solution of* *RoP*(*W* ^*i*^), *then* x* *is a Pareto Nash equilibrium of MG*.

**Proof.** The proof for this theorem directly follows from Theorem 2.2 of Hu and Mehrotra [8] and Definitions 1.1 and 2.1.

Below we describe a class of multi-objective games that can be resolved by using robust weighted approach.

**Example 1** A set of *n* manufacturers (the players) supply *P* products to *m* retailers in order to maximize the profit and minimize the risk at the same time by anticipating the order quantities of the retailers and the wholesale prices resulting from the market clearing conditions. Manufacturer *i* (*i* = 1, ⋯, *n*) chooses his supply quantity to maximize his profit and minimize the risk in the wholesale market, assuming that the other manufacturers keep their supply quantities fixed, and anticipating the market clearing wholesale market price as well as the retailer order quantities. Mathematically, manufacturer *i* faces the following multi-criteria decision problem,

$$\begin{array}{ccc} & & max\;f_{i}\left(y_{i},\pi \right):=E[\pi^{T}y_{i}-c_{i}^{T}y_{i}],\\ & & min\;r_{i}\left(y_{i},\pi \right)\\ & & s.t.\;\sum_{k=1}^{n}y_{k}=\sum_{j=1}^{m}\gamma_{j},\\ & & \;y_{i}\geq 0,\;\pi \in R^{P}, \end{array}$$ (2.2)

where *c*_*i*_ ∈ *R* ^*P*^ is the *i*th manufacturer unit uncertain cost, *y*_*i*_ ∈ *R* ^*P*^ is the supply quantity of the *i*th manufacturer and *γ*_*j*_ ∈ *R* ^*P*^ is the *j*th retailer order quantity for a given wholesale price *π*, the first constraint is the wholesale market clearing conditions, and *r*_*i*_ : *R* ^*P*^ × *R* → *R* is a general risk function for manufacturer *i*.

If each manufacturer has a set of weights $W^{i}\subset W_{r}^{i}$, *i* = 1, ⋯, *n*, then a robust weighted manufacturer equilibrium can be obtained if each manufacture solves the following robust weighted problem, *i* = 1, ⋯, *n*

$$\begin{array}{ccc} & & min\max_{w^{i}\in W^{i}}(-w_{1}^{i}f_{i}\left(y_{i},\pi \right)+w_{2}^{i}r_{i}\left(y_{i},\pi \right))\\ & & s.t.\sum_{k=1}^{n}y_{k}=\sum_{j=1}^{m}\gamma_{j},\\ & & \;y_{i}\geq 0,\;\pi \in R^{P}. \end{array}$$ (2.3)

## 3 Existence of Robust Weighted Nash Equilibrium

In this section, we first present the existence theorem on the robust weighted Nash equilibria in MG with compact and convex weight sets by using Kakutani’s [9] fixed point theorem which has been extensively used to prove the existence of an equilibrium in scalar games. We show that the robust-weighted Nash equilibria of a robust weighted multi-objective game are guaranteed to exist. Second, we discuss the conditions for ensuring the existence of robust weighted Nash equilibria. Before continuing to the existence theorem, we first give Kahutani’s fixed point theorem and a relevant definition, namely that of *upper-semi continuity*.

**Definition 3.1** *A point-to-set mapping* *ψ* : *S* → 2^*S*^ *is said to be upper semi-continuous if* *y* ^*n*^ ∈ *ψ*(*x* ^*n*^), *n* = 1, 2, 3, ⋯, $\underset{n\to \infty }{\text{lim}}x^{n}=x$, $\underset{n\to \infty }{\text{lim}}y^{n}=y$ *imply that* *y* ∈ *ψ*(*x*).

**Theorem 3.2** (Kakutani’s fixed point theorem). *Suppose that* *S* *is a closed, bounded, and convex set in Euclidean space, and* *ψ* *is an upper semi-continuous point-to-set mapping from* *S* *to the family of closed, convex subsets of* *S*, *then* ∃*x* ∈ *S* *such that* *x* ∈ *ψ*(*x*).

To utilize the above theorem, we first define a suitably constructed correspondence whose fixed point is an equilibrium. For this purpose for any given *x* = (*x* ^1^, ⋯, *x* ^*n*^) ∈ *S*, *y* = (*y* ^1^, ⋯, *y* ^*n*^) ∈ *S* and weight sets combination $W=(W^{1},\cdots ,W^{n})\subset W_{r}=(W_{r}^{1},\cdots ,W_{r}^{n})$, we define

$$\begin{array}{c} \phi^{W}\left(x\right):=\{z\in S|\;z\in arg\min_{y\in S}\rho^{W}\left(x,y\right)\}, \end{array}$$

where for any *i* ∈ *N* the function *ρ* ^*W*^ : *S* × *S* → *R* is given as follows,

$$\begin{array}{c} \rho^{W}\left(x,y\right):=\sum_{i=1}^{n}\max_{w^{i}\in W^{i}}{\left(w^{i}\right)}^{T}F^{i}\left(x^{-i},y^{i}\right). \end{array}$$

Similar to the proof of Lemma 2.3 given by [1], it is easy to see from the following lemma the equivalence of the robust weighted Nash equilibrium with the fixed point of the correspondence *ϕ* ^*W*^.

**Lemma 3.3** *A given strategy combination* $\bar{x}\in S$ *is a robust weighted Nash equilibrium of MG with* $W=(W^{1},\cdots ,W^{n})\subset W_{r}=(W_{r}^{1},\cdots ,W_{r}^{n})$ *iff* $\bar{x}$ *is a fixed point of the mapping* *ϕ* ^*W*^.

**Proof.** If $\bar{x}\in S$ is a robust weighted Nash equilibrium of MG with given $W\subset \tilde{W}$, from Definition 2.1,

$$\begin{array}{c} \max_{w_{i}\in W_{i}}{\left(w_{i}\right)}^{T}F_{i}\left({\bar{x}}_{-i},{\bar{x}}_{i}\right)\leq \max_{w_{i}\in W_{i}}{\left(w_{i}\right)}^{T}F_{i}\left({\bar{x}}_{-i},y_{i}\right),\;\forall y^{i}\in S_{i},\;i=1,\cdots ,m. \end{array}$$

The above inequality implies that

$$\begin{array}{c} \sum_{i=1}^{m}\max_{w_{i}\in W_{i}}{\left(w_{i}\right)}^{T}F_{i}\left({\bar{x}}_{-i},{\bar{x}}_{i}\right)\leq \sum_{i=1}^{m}\max_{w_{i}\in W_{i}}{\left(w_{i}\right)}^{T}F_{i}\left({\bar{x}}_{-i},y_{i}\right),\;\forall y^{i}\in S_{i},\;i=1,\cdots ,m. \end{array}$$

So from the definition of *ρ*_*W*_, $\rho_{W}(\bar{x},\bar{x})\leq \rho_{W}(\bar{x},y)$, ∀*y* ∈ *S*. Therefore $\bar{x}\in \phi_{W}(\bar{x})$, i.e., $\bar{x}$ is a fixed point of the mapping *ϕ*_*W*_.

Similarly, for any given $W=(W_{1},\cdots ,W_{m})\subset \tilde{W}=({\tilde{W}}_{1},\cdots ,{\tilde{W}}_{m})$, if $\bar{x}$ is a fixed point of the mapping *ϕ*_*W*_, we can prove that $\bar{x}\in S$ is also a robust weighted Nash equilibrium of MG with the weight set combination *W*.

The above lemma shows that the existence of the robust weighted Nash equilibria is equivalent to the existence of the fixed points of the mapping *ϕ* ^*W*^. So we only need to show that the correspondence *ϕ* ^*W*^ satisfies the assumptions of Kakutani’s theorem that is to show that for weight sets combination *W*, under some given assumptions about the functions $f_{j}^{i}(x^{-i},x^{i})$, ∀*j* = 1, ⋯, *b*_*i*_, *i* ∈ *N*, *ϕ* ^*W*^ is an upper semi-continuous point-to-set mapping from *S* to the family of closed, convex subsets of *S*. To reveal that the correspondence *ϕ* ^*W*^ meets the assumptions of Kakutani’s Theorem, we first need several technical results.

**Lemma 3.4** *Given a weight sets combination* *W* ⊂ *W*_*r*_, *if* $f_{j}^{i}(\cdot )$ *is continuous on* *S* *and for any fixed* *x* ^−*i*^, $f_{j}^{i}(x^{-i},\cdot )$ *is convex on* *S*_*i*_, ∀*j* = 1, ⋯, *b*_*i*_, *i* ∈ *N*, *then we have that*

1. *ρ* ^*W*^(⋅, ⋅) *is continuous on* *S* × *S*;
2. *for any fixed* *x* ∈ *S*, *ρ* ^*W*^(*x*, ⋅) *is convex on* *S*.

**Proof.** 1. We show that for any given *ϵ* > 0, there is a positive constant *δ* such that for any (*x*, *y*) ∈ *S* × *S* and $(\bar{x},\bar{y})\in S\times S$ if $∥(x,y)-(\bar{x},\bar{y})∥\leq \delta$ then

$$\begin{array}{c} |\rho^{W}\left(x,y\right)-\rho^{W}\left(\bar{x},\bar{y}\right)|\leq \epsilon . \end{array}$$ (3.1)

It follows from the continuity of $f_{j}^{i}(\cdot )$ that for any given *ϵ*_*i*_ > 0, there is a positive constant *δ*_*i*_ such that for any (*x*, *y*) ∈ *S*×*S* and $(\bar{x},\bar{y})\in S\times S$ if $∥(x^{-i},y^{i})-({\bar{x}}^{-i},{\bar{y}}^{i})∥\leq \delta_{i}$ then

$$\begin{array}{c} ∥F^{i}\left(x^{-i},y^{i}\right)-F^{i}\left({\bar{x}}^{-i},{\bar{y}}^{i}\right)∥\leq \epsilon_{i},\;\forall i\in N. \end{array}$$

The above inequality leads to

$$\begin{array}{ccc} & & |\max_{w^{i}\in W^{i}}{\left(w^{i}\right)}^{T}F^{i}\left(x^{-i},y^{i}\right)-\max_{w^{i}\in W^{i}}{\left(w^{i}\right)}^{T}F^{i}\left({\bar{x}}^{-i},{\bar{y}}^{i}\right)|\\ & & \leq \max_{w^{i}\in W^{i}}∥w^{i}∥∥F^{i}\left(x^{-i},y^{i}\right)-F^{i}\left({\bar{x}}^{-i},{\bar{y}}^{i}\right)∥\\ & & =∥F^{i}\left(x^{-i},y^{i}\right)-F^{i}\left({\bar{x}}^{-i},{\bar{y}}^{i}\right)∥\leq \epsilon_{i},\;\forall i\in N. \end{array}$$ (3.2)

Therefore it follows from (3.2) and

$$\begin{array}{c} ∥\left(x,y\right)-\left(\bar{x},\bar{y}\right)∥\leq \sum_{i\in N}∥\left(x^{-i},y^{i}\right)-\left({\bar{x}}^{-i},{\bar{y}}^{i}\right)∥ \end{array}$$

that given $\epsilon :=\sum_{i\in N}\epsilon_{i}$, there is $\delta :=\sum_{i\in N}\delta_{i}$ such that $∥(x,y)-(\bar{x},\bar{y})∥\leq \delta$ and (3.1) holds.

2. For any fixed *x* ∈ *S*, given *λ* ∈ [0, 1] and $y,\;\bar{y}\in S$, we have that

$$\begin{array}{ccc} & & \rho^{W}\left(x,\lambda y+\left(1-\lambda \right)\bar{y}\right)=\sum_{i=1}^{n}\max_{w^{i}\in W^{i}}{\left(w^{i}\right)}^{T}F^{i}\left(x^{-i},\lambda y^{i}+\left(1-\lambda \right){\bar{y}}^{i}\right)\\ & & \leq \sum_{i=1}^{n}\max_{w^{i}\in W^{i}}{\left(w^{i}\right)}^{T}(\lambda F^{i}\left(x^{-i},y^{i}\right)+\left(1-\lambda \right)F^{i}\left(x^{-i},{\bar{y}}^{i}\right))\\ & & \leq \lambda \sum_{i=1}^{n}\max_{w^{i}\in W^{i}}{\left(w^{i}\right)}^{T}F^{i}\left(x^{-i},y^{i}\right)+\left(1-\lambda \right)\sum_{i=1}^{n}\max_{w^{i}\in W^{i}}{\left(w^{i}\right)}^{T}F^{i}\left(x^{-i},{\bar{y}}^{i}\right)\\ & & =\lambda \rho^{W}\left(x,y\right)+\left(1-\lambda \right)\rho^{W}\left(x,\bar{y}\right), \end{array}$$

where the first inequality comes from the convexity of $f_{j}^{i}(x^{-i},\cdot )$.

The above lemma gives the continuity and convexity for function *ρ* ^*W*^ which are two key results for proving the main existence theorem below. The two results are used to prove the upper semi-continuity and convexity of the mapping *ϕ* ^*W*^ respectively. We now propose the main result of this section.

**Theorem 3.5** *Suppose each strategy set* *S*_*i*_ *is a nonempty compact convex subset of* $R^{a_{i}}$, ∀*i* ∈ *N*. *Then under the conditions of Lemma 3.4, the multi-objective game* *MG* *has at least one robust weighted Nash equilibrium with* *W* ⊂ *W*_*r*_.

**Proof.** From Lemma 3.3, for the proof of this theorem, it suffices to show that the correspondence *ϕ* ^*W*^ meets the assumptions of Kakutani’s theorem. To this end, we need to show that *ϕ* ^*W*^ is an upper semi-continuous point-to-set mapping from *S* to the family of closed, convex subsets of *S*. We first show that for any given *x* ∈ *S*, *ϕ* ^*W*^(*x*) is a closed, convex subset of *S*.

From the continuity of *ρ* ^*W*^(⋅, ⋅) and the existence of the minimum of the continuous function on a compact set, we have that $arg\underset{y\in S}{\text{min}}\rho^{W}(x,y)\neq \emptyset$. Therefore *ϕ* ^*W*^(*x*) ≠ ∅, for any *x* ∈ *S*. Note that by definition, *ϕ* ^*W*^(*x*) ∈ *S*, ∀*x* ∈ *S*. Next we show that *ϕ* ^*W*^(⋅) is convex for any *x* ∈ *S*. Suppose *z* : = (*z* ^1^, ⋯, *z* ^*n*^), *v* : = (*v* ^1^, ⋯, *v* ^*n*^) ∈ *ϕ*(*x*). Then for any *λ* ∈ [0, 1], we have

$$\begin{array}{c} \rho^{W}\left(x,\lambda z+\left(1-\lambda \right)v\right)\leq \lambda \rho^{W}\left(x,z\right)+\left(1-\lambda \right)\rho^{W}\left(x,v\right)\leq \rho^{W}\left(x,y\right),\;\forall y\in S, \end{array}$$ (3.3)

where the first inequality comes from the convexity of *ρ* ^*W*^(*x*, ⋅) and the second inequality comes from the definitions for *z* and *v*. The above inequality and the convexity of *S* lead to *λz* + (1−*λ*)*v* ∈ *ϕ* ^*W*^(*x*) which implies that *ϕ* ^*W*^(⋅) is convex for any *x* ∈ *S*.

Finally, we show that *ϕ* ^*W*^(⋅) is a upper semi-continuous correspondence. Suppose *x*_*n*_ → *x*, *z*_*n*_ → *z*, and *z*_*n*_ ∈ *ϕ* ^*W*^(*x*_*n*_), then we have

$$\begin{array}{c} \rho^{W}\left(x_{n},z_{n}\right)\leq \rho^{W}\left(x_{n},y\right),\;\forall y\in S. \end{array}$$

Taking limits in the above inequality and noting the continuity of *ρ* ^*W*^(⋅, ⋅), we have

$$\begin{array}{c} \rho^{W}\left(x,z\right)\leq \rho^{W}\left(x,y\right),\;\forall y\in S. \end{array}$$

The above inequality and the compactness of set *S* imply that *z* ∈ *ϕ* ^*W*^(*x*). Therefore, we complete the proof that *ϕ* ^*W*^ is an upper semi-continuous correspondence and the closeness of the set *ϕ* ^*W*^ for any *x* ∈ *S* follows from the upper-semi-continuity of *ϕ*. Therefore, *ϕ* ^*W*^ meets the assumptions of Kakutani’s fixed point theorem.

## 4 Conclusion

A robust weighted approach is proposed to a class of multi-objective games, where each player has a set of weights to its objectives instead of accessing accurate weights. This method leads to the corresponding robust weighted game model and a robust weighted Nash equilibrium is presented to address the new model. We show the existence of equilibrium under mild assumptions about the multi-objective payoffs. Further studies are to see how to design appropriate algorithms so as to numerically realize the robust weighted Nash equilibrium.
